# Derivative 11;22 (Emanuel) Syndrome: A Case Report and A Review

**DOI:** 10.1155/2013/237935

**Published:** 2013-04-18

**Authors:** Madan Gopal Choudhary, Prashant Babaji, Nitin Sharma, Dilip Dhamankar, Gururaj Naregal, Vijay Sunil Reddy

**Affiliations:** ^1^Department of Pediatrics, SPMC Medical College, Bikaner, Rajasthan 334001, India; ^2^Department of Pedodontics, SPPIDMS, Lucknow 226001, India; ^3^Department of Pedodontics, Vyas Dental College, Jodhpur, Rajasthan 342001, India; ^4^Department of Prasthododntics, NIMS Dental College, Jaipur 302001, India; ^5^Department of Periodontics, SJM Dental College, Chitradurga 577501, India; ^6^Department of Periodontics, College of Dental Science, Bhavnagar 364001, India

## Abstract

Emanuel syndrome (ES) is a rare anomaly characterized by a distinctive phenotype, consisting of characteristic facial dysmorphism, microcephaly, severe mental retardation, developmental delay, renal anomalies, congenital cardiac defects, and genital anomalies in boys. Here, we report a male neonate, with the classical features of Emanuel syndrome.

## 1. Introduction 

 Emanuel syndrome (ES) is an unbalanced translocation syndrome usually arises through a 3 : 1 meiosis I malsegregation during gametogenesis in a balanced translocation phenotypically normal carrier [1]. Patients with Emanuel syndrome have a distinctive phenotype, which consists of characteristic facial dysmorphism, microcephaly, severe mental retardation, delay in developmental milestone, renal anomalies, congenital cardiac defects, and genital anomalies in boys [2]. While the true mortality rate in Emanuel syndrome is unknown, long-term survival is possible [3]. Emanuel syndrome is also referred to as derivative 22 syndrome, derivative 11;22 syndrome, partial trisomy 11;22, or supernumerary der (22)t(11;22) syndrome [2, 4, 5]. 

## 2. Case Report

**Figure 1:**
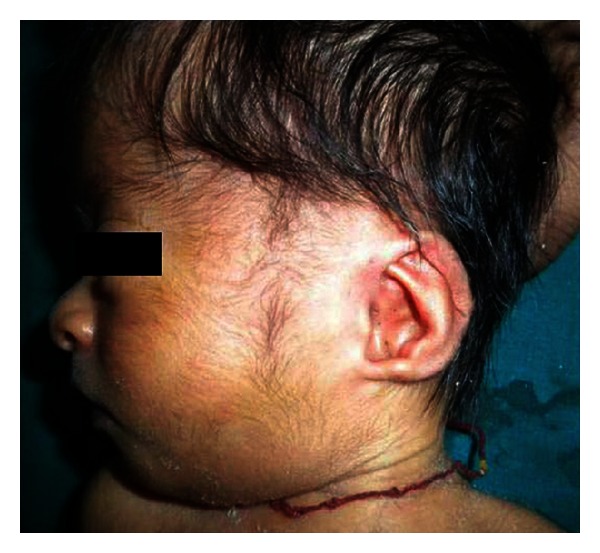
The photograph shows the facial features with downslanting palpebral fissure, large and low-set ears with preauricular pit, and micrognathia.

**Figure 2:**
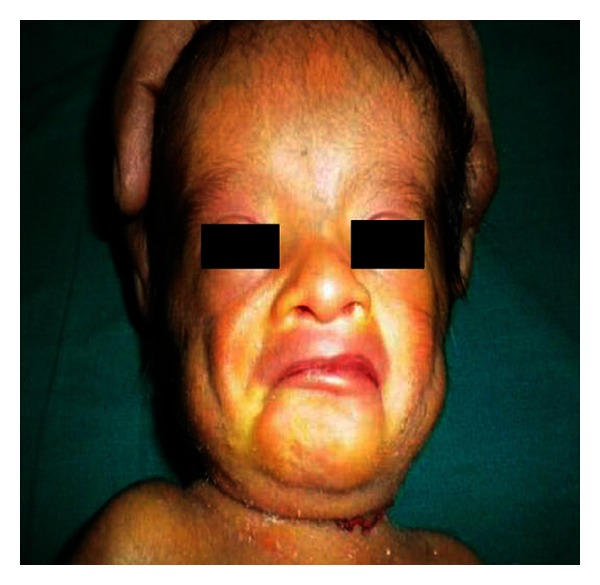
The photograph shows the facial features with prominent forehead, widely separated eyes with downslanting palpebral fissure, and micrognathia.

 A young mother aged 22 years was reported with a male neonate. The marriage of the infant's parents was consanguineous. The antenatal period of the infant was uneventful except relative less-marked abdominal enlargement and less perception of fetal movements. The infant was delivered at full term by vaginal delivery. On examination, he was small for gestational age as the birth weight was 2.2 kg (<third percentile), length was 46 cm (<third percentile), and head circumference was 32 cm (<third percentile). He had a remarkable facial appearance which included prominent forehead with dilated veins, widely separated eyes with downslanting palpebral fissure, broad nasal bridge, prominent philtrum, bilateral large and low-set ears with preauricular pit (Figures 1 and 2). He was also having a small penis (1.5 cm), but both testes were completely descended. Oral findings observed were high arched palate and micrognathia. 

**Figure 3:**
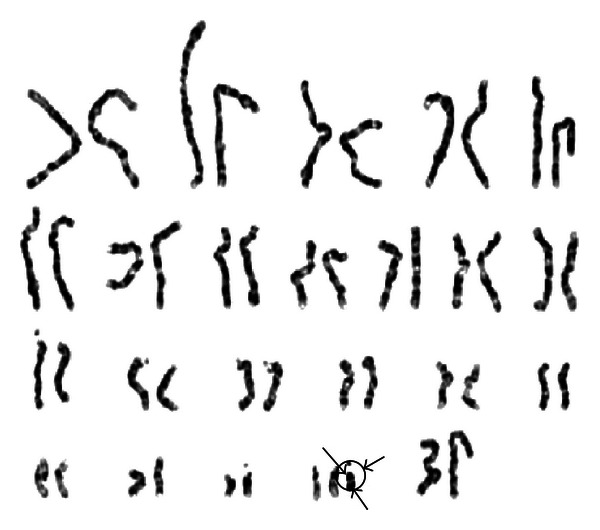
The patient's karyotype shows an extra supernumerary chromosome.

**Figure 4:**
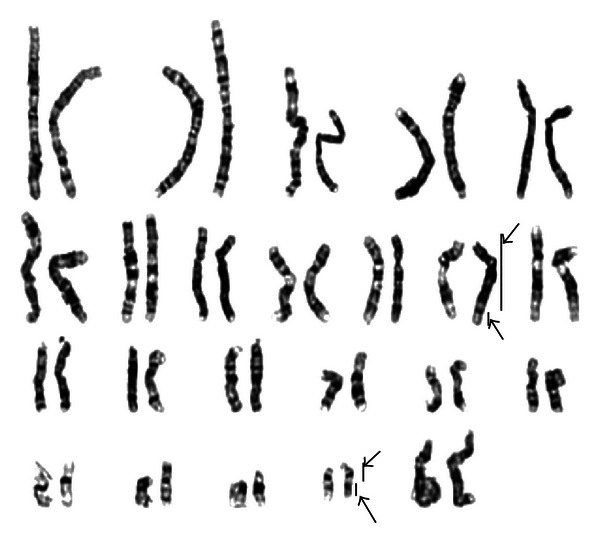
His mother's karyotype shows a balanced non-Robertsonian translocation between chromosome 11 and chromosome 22.

 Echocardiography revealed a moderately large, subaortic ventricular septal defect (VSD). The right kidney was missing on abdominal ultrasonography. Hearing assessment revealed a mild hearing loss, but ophthalmological assessment was unremarkable. Karyotyping using G-banding analysis at 550 band levels showed an extra supernumerary marker chromosome (SMC) with supernumerary derivative (22)t(11;22) (Figure 3). To ascertain the origin of this SMC, karyotyping for his parents was performed. The mother was found to be a balanced carrier; 46,XX,t(11;22)(q23.3;q11.2) (Figure 4). During the followup examination for 3 years, he was found to have a significant central hypotonia and developmental delay, and all growth parameters remained well below the third percentile. On followup examinations, he showed developmental delay, and all growth parameters remained well below the third percentile at six months of age. 

## 3. Discussion 

 Emanuel syndrome is an inherited chromosomal abnormality syndrome [1, 14]. Supernumerary marker chromosomes (SMCs) are frequent findings in cytogenetic studies, with 9% of SMCs derived from chromosome 22 [15]. This chromosome imbalance consists of either a derivative chromosome 22 [der(22)] as a supernumerary chromosome with the following karyotype: 47,XX,+der(22)t(11;22)(q23;q11) in females or 47,XY,+der(22)t(11;22)(q23;q11) in males rarely [3]. It was named as Emanuel syndrome in 2004 (OMIM no. 609029) [3, 5]. 

 The exact incidence is unknown. This is a rare syndrome with reported cases of around 100.  Table 1 shows the various reported cases found on Google, PubMed/MEDLINE search [6–13]. Male and female balanced carriers have 0.7% and 3.7% risk of having children with supernumerary der(22), respectively [4]. Patients with ES has a distinctive phenotype, consisting of characteristic facial dysmorphism including prominent forehead, epicanthal folds, downslanting palpebral fissures, broad and flat nasal bridge, long and pronounced philtrum, abnormal auricles ranging from microtia to large ears often associated with a preauricular ear pit and/or skin tags, microcephaly, severe mental retardation, developmental delay, renal anomalies, congenital cardiac defects, and genital anomalies in boys. Oral findings commonly are micrognathia, cleft, or high-arched palate [2, 3]. Evolution of facial dysmorphism with age is not well described, but Medne et al. in 2007 suggested that facial features of ES coarsen over time with micrognathia becoming less pronounced [16]. Almost all the children with ES have global developmental delay and intellectual disability. While most children do not independently ambulate, over 70% of subjects eventually learned to walk with support. Expressive language is significantly impaired, with rudimentary speech acquisition in only 20% [3].  Table 2 shows the list of clinical features observed in Emanuel syndrome [3, 4, 14, 16].

 The most important differential diagnosis of Emanuel syndrome is the cat eye syndrome (CES). CES usually results from partial tetrasomy 22. Iris coloboma, however, which is a cardinal feature of CES, is not reported in ES. Unlike ES, the majority of individuals with CES have mild or no intellectual impairment [17]. Other differential findings can be Fryns syndrome, Smith-Lemli-Opitz syndrome, or Kabuki syndrome [16]. Clinical testings like chromosomal analysis, FISH testing, whole chromosome paint (WCP), array genomic hybridization (aGH), or MLPA assay can be performed for the diagnosis of this syndrome [14, 18, 19]. 

 Management involves multidisciplinary team approach involving pedodontist, pediatrician, plastic surgeon, geneticist, gastrologist, speech therapist, urologist, cardiologist, ENT surgeon, and ophthalmologist. Patients with cleft palate have feeding difficulties, which requires feeding plate and surgical closure of cleft palate. The long-term prognosis is directly related to the associated congenital malformations. Highest mortality is in the first few months of life. While the true mortality rate in ES is unknown, long-term survival is possible, especially if the patient survives infancy period [3]. The reported case had all the classical features of ES. 

 Two issues are important in terms of genetic counseling of these families. First, when one parent is a carrier of t(11;22), future pregnancies are at an increased risk for either ES, balanced t(11;22), or another meiotic malsegregation, so prenatal cytogenetic testing should be offered in future pregnancies. Secondly, carrier testing of the unaffected siblings should normally be offered when they have reached adulthood and are able to understand the reproductive implications of being a carrier. 

## 4. Conclusion 

 It is necessary to emphasize the importance of suspecting this syndrome, if a neonate presents with the foresaid facial dysmorphic features and congenital anomalies, so that early diagnosis and timely intervention can be taken in an effort to prolong the survival and improve the lifestyle and more importantly to give appropriate advice regarding genetic counseling to family members. 

## Figures and Tables

**Table 1:** List of reported cases of Emanuel syndrome [6–13].

Sl. no.	Reference	Year	No. of cases reported
1	Zaki et al.	2012	1
2	Walfisch et al.	2012	5
3	Kim et al.	2012	1
4	Carter et al.	2009	63
5	Toyoshima et al.	2009	1
6	Emanuel	2008	1
7	Prieto et al.	2007	1
8	Crolla et al.	2005	1
9	Hou	2003	1
10	Rosias et al.	2001	1
11	Estop et al.	1999	1
12	Funke et al.	1999	1
13	Shaikh et al.	1999	1
14	Dawson et al.	1996	1
15	Beedgen et al.	1986	1
16	Fraccaro et al.	1980	1
17	Kessel and Pfeifer	1977	1

**Table 2:** List of clinical features observed in Emanuel syndrome [3, 4, 14, 16].

Sl. no.	System involved	Clinical features of Emanuel syndrome
1	Growth and development	Pre and postnatal growth retardation, delayed speech, and language development (more commonly)

2	Craniofacial	Microbrachycephaly, prominent forehead, epicanthal folds, downslanting palpebral fissures, broad and flat nasal bridge, long pronounced philtrum, abnormal auricles, preauricular ear pits and/or tags 76%, deafness, and otitis media

3	CNS	Microcephaly present most commonly, seizures, failure to thrive, and delayed pschomotor development

4	Cardiac	60% individuals with congenital heart defects like atrial septal defect, ventricular septal defect, Tetralogy of Fallot, and patent ductus arteriosus

5	Genitointestinal	Diaphragmatic hernia, anal atresia, inguinal hernias, biliary atresia, small penis 64%, and cryptorchidism 46%

6	Musculoskeletal	Centrally based hypotonia most commonly, congenital hip dislocation, arachnodactyly, club foot and joint, syndactyly of the toes, delayed bone age, and hyperextensibility of joints

7	Oral findings	Cleft palate 50%, micrognathia 60%, angular mouth pits, bifid uvula, and facial asymmetry

8	Immunological	Congenital immunological deficiency

9	Renal	Renal defects 36%
